# The Alcohol Intervention Training Program (AITP): A response to alcohol misuse in the farming community

**DOI:** 10.1186/1471-2458-11-242

**Published:** 2011-04-19

**Authors:** Susan A Brumby, Alison J Kennedy, David Mellor, Marita P McCabe, Lina A Ricciardelli, Alexandra Head, Catherine Mercer-Grant

**Affiliations:** 1 School of Medicine, Deakin University, National Centre for Farmer Health (NCFH), PO Box 283, Hamilton VIC 3300, Australia; 2 Western District Health Service, PO Box 283, Hamilton VIC 3300, Australia; 3 School of Psychology, Deakin University, 221 Burwood Hwy, Burwood VIC 3125, Australia

## Abstract

**Background:**

Farm men and women in Australia have higher levels of problematic alcohol use than their urban counterparts and experience elevated health risks associated with excessive alcohol consumption. The Sustainable Farm Families (SFF) program has worked successfully with farm men and women to address health, well- being and safety and has identified that further research and training is required to understand and address alcohol misuse behaviours. This project will add an innovative component to the program by training health professionals working with farm men and women to discuss and respond to alcohol-related physical and mental health problems.

**Methods/Design:**

A mixed method design with multi-level evaluation will be implemented following the development and delivery of a training program (The Alcohol Intervention Training Program {AITP}) for Sustainable Farm Families health professionals. Pre-, post- and follow-up surveys will be used to assess both the impact of the training on the knowledge, confidence and skills of the health professionals to work with alcohol misuse and associated problems, and the impact of the training on the attitudes, behaviour and mental health of farm men and women who participate in the SFF project. Evaluations will take a range of forms including self-rated outcome measures and interviews.

**Discussion:**

The success of this project will enhance the health and well-being of a critical population, the farm men and women of Australia, by producing an evidence-based strategy to assist them to adopt more positive alcohol-related behaviours that will lead to better physical and mental health.

## Background

Compared to their urban counter-parts, members of Australian rural communities, particularly farm men and women, are more likely to experience a range of negative health outcomes [1,2]. One area identified as a major problem for farm communities is alcohol misuse and its associated problems - preliminary research suggests that 54 per cent of men and 22 per cent of women in the broad acre agriculture industry engage in high risk drinking at least monthly [3,4] based on the National Health and Medical Research Council (NHMRC) 2001 guidelines [5]. The Western District Health Service's (WDHS) Sustainable Farm Families (SFF) program works with farm men and women to educate them about health issues and to increase positive health behaviours. The program consists of a structured two-day workshop in year one and a one-day workshop in years two and three. While the SFF has produced gains in many domains, a preliminary (unpublished) survey that our research team conducted highlighted that SFF health professionals felt they lacked the knowledge, confidence and skills required to work with alcohol-related problems in their communities. Our pilot work demonstrated that most SFF health professionals felt it was much easier to discuss behavioural risk factors such as obesity, smoking and lack of exercise than drinking too much. In line with this, and whilst 75% of the 45 health professionals surveyed indicated that they knew where to refer people with alcohol problems, there were only three alcohol related referrals documented.

In this project, SFF health professionals will be trained to recognise alcohol misuse, develop skills to overcome the barriers to discussing alcohol problems and to deliver an initial brief intervention. Brief interventions have been found to be effective in reducing the severity of dependence and alcohol-related problems, even among people with harmful drinking levels [6,7].

The aim of this paper is to describe the development and proposed evaluation of the Alcohol Intervention Training Program (AITP) - a training program designed to equip these health professionals to address alcohol misuse and its associated problems in farm men and women.

## Methods/Design

### Target professionals

The target population will be health professionals, who are registered nurses (Division 1) trained to deliver the SFF program to farm men and women within their own and other farming communities. These nurses have expertise in rural health, and men's and women's health, and experience working with farm men and women. In addition to their active involvement in the SFF program, these health professionals also work in areas ranging from community health to acute care settings.

### Comparison groups

A sample size of 100 was calculated, based on a medium effect size, to establish a power level above 95%[8]. The health professionals involved in the delivery of SFF programs will be allocated to either a) the AITP trained intervention group, or b) the wait list control group (no intervention apart from the collection of outcome data) who will be offered the training at a later date. All attempts will be made to ensure numbers will be even between groups.

### Intervention design

The evaluation design will be quasi-experimental that compares the intervention and control groups over four key areas at three time points. The timeline is outlined in Table 1.

**Table 1:** Evaluation timeline for the Alcohol Intervention Training Program (AITP)

Alcohol Intervention Training Program outcome tools for SFF health professionals	Pre-training	Post-training	3 month follow-up
1. Knowledge of alcohol misuse	✓	✓	✓

2. Willingness to work with people who misuse alcohol	✓	✓	✓

3. Self-efficacy when working with people who misuse alcohol	✓	✓	✓

4. Perceived barriers to working with people who misuse alcohol	✓	✓	✓

### Intervention material

The intervention material was initially developed from the researchers' experience in the drug and alcohol and mental health fields. The program was tailored to be relevant to the rural sector through the researchers' experience working with farm men and women. The Alcohol Intervention Training Program (AITP) has been designed to be delivered in four sessions over two days and the sessions are outlined in Table 2. A presenter's manual and slide presentation has been developed and will be accompanied by a training support kit for participants.

**Table 2:** Delivery format and content of the Alcohol Intervention Training Program (AITP)

Session 1	Session 2	Session 3	Session 4
Understanding alcohol misuse	Detecting and assessing alcohol use problems*	Communication Skills	Brief Interventions
• diagnostic definitions	Training in the use of the:	• dual relationships	• blood alcohol concentration (BAC)
• NHMRC guidelines for safe drinking [9]	• Alcohol Use Disorders Intervention Test (AUDIT)[10]	• active listening • probing techniques	• personal responsibility for change
• physiological, psychological, social and financial effects of alcohol misuse.	• Short Index of Problems (SIP)[11] • Depression, Anxiety and Stress Scale (DASS21)[12]	• motivational interviewing	• methods for employing empathy and supporting self-efficacy
	• Readiness To Change Questionnaire (RTCQ)[13].		• training in the use of the Timeline follow back (TLFB) [14]drinking calendar.

* These screening and assessment tools specifically target alcohol misuse and associated mental health issues and are widely used in research and clinical settings. These measures will also be used as outcome measures to evaluate changes in the alcohol use and mental health of farm men and women with whom SFF health professionals work

### AITP training support kit

This kit will include a plain language statement and consent form; pre and post-training questionnaires; a copy of the slides; a set of worksheets containing group exercises, assessment and screening tools and a range of case scenarios to which the tools could be applied; a course evaluation form; and, a post-training information support package. The information support package will contain laminated standard drink information cards and standard drink measures tumbler; background material on the screening and assessment tools; and pamphlets covering the National Centre for Farmer Health (NCFH) website, alcohol and mental health referral services, further information resources on alcohol and mental health and tips for reducing drinking alcohol.

### Process evaluation

The initial stage of process evaluation will involve a pilot implementation of the AITP with two groups of three experienced health professionals. These health professionals have had extensive experience with the SFF program but are no longer active presenters of the material. Pilot participants will provide extensive verbal and written feedback both during and after the training that will be used to inform the refinement of the AITP. During the implementation of the AITP with the intervention group, written evaluations and feedback will also be sought from health professionals. This feedback will be gathered following each of the four sessions comprising the AITP as outlined in Table 2.

### Impact and outcome evaluation

#### Evaluation measures for SFF health professionals

Assessments using the following evaluation measures for health professionals (intervention and control groups) will be completed pre- and post-training, as well as at a three month follow-up (refer to Table 1):

1. *Knowledge of alcohol misuse questionnaire*. This multiple choice questionnaire was developed by the research team as a means of assessing the health professionals general knowledge about alcohol and alcohol misuse and contains 12 questions relating to material covered in the AITP training manual. The questions were determined within a number of brainstorming sessions by the research team.

2. *SAAPPQ (Short Alcohol and Alcohol Problems Perception Questionnaire)*. This questionnaire comprises two subscales from the Alcohol and Alcohol Problems Perception Questionnaire (AAPPQ) [15] and was used to determine the health professionals' willingness to work with clients who have alcohol problems. This scale has been reliably used in a number of studies [16-18]

3. *Self-efficacy scale*. This scale was adapted from a scale originally developed to assess the self-efficacy of care staff in working with depression in the aged care sector. It assesses the level of confidence health professionals have in their ability to work with the alcohol problems of farm men and women [18].

4. *Perceived barriers to working with alcohol problems scale*. This scale assesses perceived barriers to working with alcohol-related problems and was adapted from a scale developed by the authors for assessing perceived barriers when working with depression [19].

Semi-structured interviews will be conducted with each of the health professionals who have participated in the training program to obtain their views on the program and how it impacts or is anticipated to impact their work with the SFF program and their other work in the rural health sector. This interview process will develop the detail of the data gathered in questionnaire format and endeavour to extract more specific information about health professional reactions to the training. Particular focus will be on any barriers to using and implementing the training material and on suggestions for change and follow-up support requirements. The data gained from the interviews will be used to amend the AITP for future training delivery.

### Outcome measures for farm men and women

Prior to the commencement of the intervention we will collect baseline data from 400 farm men and women participating in the SFF program on alcohol-related behaviour and mental health. The sample size was calculated, based on a medium effect size, to achieve a power level of over 85% [8]. This data will be collected in both the intervention and control groups. This assessment will be used to identify those farm men and women who have alcohol-related problems and associated mental health problems. Based on previous findings [1,3]we estimate that this number will be approximately 55% of farm men and 25% of farm women. Following the implementation of the AITP training program, we will re-assess the alcohol-related behaviour and mental health of farm men and women to establish whether those working with SFF health professionals who have undertaken our training program have outcomes superior to those who work with health professionals who have not undertaken our program. The outcome timeline and measures for farm men and women are outlined in Table 3.

**Table 3:** Evaluation timeline for farm men and women

Evaluation measures for farm men and women	Baseline at first SFF workshop	Follow-up at second SFF workshop
Alcohol use and its impact (AUDIT [10], SIP [11])	✓	✓

Mental health (DASS21[12])	✓	✓

Readiness to change (RTCQ [13])	✓	✓

### Outcome measures for farm men and women

#### Alcohol use and its impact

The Alcohol Use and Disorders and Identification Test (AUDIT), developed by the World Health Organisation (WHO) is an international test for early identification of hazardous and harmful alcohol use. The instrument consists of 3 subscales: alcohol consumption, dependence and harm. It has been widely use in alcohol-related research and alcohol-related health problems [20,21].

The Short Index of Problems (SIP) [11], a brief version of the Drinker Inventory of Consequences (DrInC)[11] which was designed to assess 5 alcohol-related problem areas: physical, intrapersonal, social responsibility, interpersonal, and impulse control. The brief version has 15 questions. Miller et al. [11] reported an internal consistency of 0.81, and Feinn et al. [22] obtained an internal consistency of 0.79.

#### Mental Health

The DASS21, was used to measure the three dimensions specified in Lovibond and Lovibond's [12] tripartite model of affect: low positive affect (Depression), physiological hyper arousal (Anxiety), and negative affect (Stress). Each dimension is assessed by a seven-item subscale. The psychometric properties of the original 42-item version of the DASS are well established [23], and the short form maintains these properties [24,25].

#### Readiness to Change

Participant's intention to change their drinking behaviour will be assessed using the Readiness to Change Questionnaire [13]. This instrument is based on the stages of change model of Prochaska, DiClemete and Norcross [26] and assigns participants to one of three stages of change: precontemplation, contemplation or action. The measure has demonstrated good internal consistency and test-retest reliability, and strong predictive validity [27,28].

We will also conduct interviews with 15 farm men and women who have received services from AITP trained SFF health professionals and 15 farm men and women who have received services from untrained SFF health professionals, with a focus on their experiences related to discussions, assessment and interventions for alcohol-related problems (Table 4).

**Table 4:** Sequence of the intended outcomes from the AITP over time

Participation in the AITP	Changes in outcome measures of SFF health professionals	Behaviour changes in SFF health professionals	Changes in outcome measures of farm men and women	Behaviour changes in farm men and women
				
	Measured pre and post-training and at a three month follow-up	Measured at post-training interviews and post-SFF program contact with farm men and women	Measured at baseline and post-SFF program contact with health professionals	Projected changes

	-Knowledge of alcohol misuse -Willingness to work with people who misuse alcohol -Self-efficacy when working with people who misuse alcohol -Perceived barriers to working with people who misuse alcohol	-Increased recognition of alcohol misuse -Usage and anticipated usage of the AITP material -Increased referral rates for alcohol-related problems	-Alcohol use and its impact (AUDIT [10] and SIP [11]) -Mental health (DASS21[12]) -Readiness to change (RTCQ [13])	-Reduced levels of alcohol misuse -Improved mental health -Reduced risk of associated physical harm -Reduced risk of associated social and financial harm

### Data analysis

The effectiveness of the AITP will be evaluated using repeated measures analysis of variance using both the outcome measures from the health professionals and the farm men and women.

All qualitative interviews will be recorded, transcribed and double-checked for accuracy. Initially, the research team will work individually and then collaboratively using a modified grounded theory approach [29]. Given the small sample size and the nature of the interview material, the principals of interpretative phenomenological analysis will be adopted [30]. The research team will analyse a cross-section of the interviews to identify and interpret the key themes arising. All of the interviews will then be individually dissected and the extracts sorted into these themes.

### Consent and Ethics

All participating health professionals will be provided with a plain language statement and will provide informed written consent. Various strategies will be used to maximise the response rate of participants. The project has been approved by the Deakin University Human Ethics Advisory Group - Faculty of Health, Medicine, Nursing, and Behavioural Sciences (HEAG-H 182/09).

## Discussion

The problems associated with alcohol misuse in farming communities continue to demand attention. The National Rural Health Alliance describe farming as "one of the riskiest occupations in terms of injury rates both in Australia and overseas" (p.15)[31]. Alcohol seems to be consistently linked to the risks of rural life. Nearly 50% of all road crashes take place on open rural roads [31]. Alcohol was found to be involved in approximately 18% of rural and remote crashes and 30% of fatal rural and remote crashes reported by Queensland police [32]. Links between farm related fatalities and alcohol have also been identified with 13.3% of 338 fatally injured persons tested being found to have a BAC over .05 [33]. High risk drinking and alcohol-related mortality continue to be at higher levels for rural dwellers, particularly among young adults, when compared with their urban counterparts [34]. The economic cost estimates of alcohol misuse in Australia, recently increased to $36 billion [35], compound the health and social costs of excessive alcohol consumption. Our project will add an important component to the SFF program by producing an evidence-based strategy for encouraging farm men and women to adopt behaviours that will reduce alcohol-related problems and promote better physical and mental health. The knowledge generated from this project will add significantly to the evidence base and can shape future training and interventions both nationally and internationally.

## Competing interests

The authors declare that they have no competing interests.

## Authors' contributions

AK drafted the initial manuscript. SB developed the Sustainable Farm Families model, and had significant input with compilation of the manuscript. CMG oversaw participation of the health professionals and participation of farmers. DM, LAR, MPM, SB and CMG contributed to the methods and design of the study. All authors have had critical input into the production of the final manuscript and have read and approve of the final manuscript.

## Pre-publication history

The pre-publication history for this paper can be accessed here:

http://www.biomedcentral.com/1471-2458/11/242/prepub
